# Genome-Wide Changes of Regulatory Non-Coding RNAs Reveal Pollen Development Initiated at Ecodormancy in Peach

**DOI:** 10.3389/fmolb.2021.612881

**Published:** 2021-04-09

**Authors:** Jiali Yu, Dennis Bennett, Christopher Dardick, Tetyana Zhebentyayeva, Albert G. Abbott, Zongrang Liu, Margaret E. Staton

**Affiliations:** ^1^ Genome Science and Technology Program, University of Tennessee, Knoxville, TN, United States; ^2^ Appalachian Fruit Research Station, United States Department of Agriculture—Agriculture Research Service, Kearneysville, WV, United States; ^3^ Department of Ecosystem Science and Management, Schatz Center for Tree Molecular Genetics, The Pennsylvania State University, University Park, PA, United States; ^4^ Forest Health Research and Education Center, University of Kentucky, Lexington, KY, United States; ^5^ Department of Entomology and Plant Pathology, Institute of Agriculture, University of Tennessee, Knoxville, TN, United States

**Keywords:** dormancy, microRNA, lncRNA, siRNA, *Prunus persica*

## Abstract

Bud dormancy is under the regulation of complex mechanisms including genetic and epigenetic factors. To study the function of regulatory non-coding RNAs in winter dormancy release, we analyzed the small RNA and long non-coding RNA (lncRNA) expression from peach (*Prunus persica*) floral buds in endodormancy, ecodormancy and bud break stages. Small RNAs underwent a major shift in expression primarily between dormancy and flowering with specific pairs of microRNAs and their mRNA target genes undergoing coordinated differential expression. From endodormancy to ecodormancy, ppe-miR6285 was significantly upregulated while its target gene, an ASPARAGINE-RICH PROTEIN involved in the regulation of abscisic acid signaling, was downregulated. At ecodormancy, ppe-miR2275, a homolog of meiosis-specific miR2275 across angiosperms, was significantly upregulated, supporting microsporogenesis in anthers at a late stage of dormancy. The expression of 785 lncRNAs, unlike the overall expression pattern in the small RNAs, demonstrated distinctive expression signatures across all dormancy and flowering stages. We predicted that a subset of lncRNAs were targets of microRNAs and found 18 lncRNA/microRNA target pairs with both differentially expressed across time points. The genome-wide differential expression and network analysis of non-coding RNAs and mRNAs from the same tissues provide new candidate loci for dormancy regulation and suggest complex noncoding RNA interactions control transcriptional regulation across these key developmental time points.

## Introduction

Perennial plants from temperate regions complete an annual life cycle of growth and dormancy. To survive unfavorable environmental conditions in winter, plants enter dormancy, which protects their reproductive tissues from freezing temperatures. Bud dormancy has been defined as three stages: endodormancy, ecodormancy, and paradormancy (Lang et al., 1987). In the endodormant and ecodormant states, the buds do not exhibit external visible growth. To release from endodormancy, buds have to be exposed to a period of chill, usually measured in hours of 0–7°C and referred to as chill requirement (CR) or chill hours (CH). Once the CR is fulfilled, buds transit from endodormancy to ecodormancy, when buds remain dormant while cold temperatures persist, but will reactivate their growth as soon as favorable conditions occur. Thus, dormancy is also a strategy to synchronize vegetative bud break or flowering to the optimal climate conditions in a year (Ding and Nilsson, 2016). With climate change, plants experiencing warm winters are impacted if they do not experience sufficient CR to fully release from dormancy, which impacts reproduction (Luedeling et al., 2011; Bartolini et al., 2020). Alternatively, earlier warm spells during winter or early spring may induce early flowering, then subsequent frosts can damage newly emerged tissues (Viti et al., 2010; Martínez-Gómez et al., 2017). These losses make dormancy research important both economically for fruit and nut trees and ecologically for forest trees. Despite decades of research, the underlying mechanisms controlling dormancy transition and release in temperate trees and other perennial species are still unclear.

Many studies on the mechanisms of dormancy regulation have been focused on dormancy related transcription factors (da Silveira Falavigna et al., 2018), hormone signals (Liu and Sherif, 2019), oxidative stress (Beauvieux et al., 2018), plasmodesmata and the plasma membrane (Rinne et al., 2011; Tylewicz et al., 2018), phenylpropanoid (Conrad et al., 2019) and carbohydrate metabolism (Park et al., 2009; Kaufmann and Blanke, 2017; Signorelli et al., 2018). The transition from dormancy to bud break is a complex process that integrates transcriptional regulation, metabolic signaling and chromatin modification (Lloret et al., 2018; Singh et al., 2018). Our previous transcriptome study in apricot and peach during dormancy progression identified genes enriched in epigenetic regulation pathways including histone modification, RNA interference, and production of small RNAs involved in gene silencing (Yu et al., 2020). Studies in other species have also identified epigenetic regulation of dormancy. Changes in DNA methylation and small interfering RNAs were observed in sweet cherry during bud dormancy (Rothkegel et al., 2017). Long non-coding RNAs and small RNAs were also reported to be involved in the regulation of vernalization and cold acclimation in more phylogenetically distant species, including Arabidopsis and Norway spruce (Heo and Sung, 2011; Yakovlev et al., 2011; Kim and Sung, 2017; Tian et al., 2019).

Small RNAs are short non-coding RNAs ranging from 20 to 24 nucleotides (nt) and many are involved in abiotic and biotic stress response, genome reprogramming and plant development. They are known to function as regulators of gene expression, either directly by suppressing gene translation or indirectly by silencing expression via chromatin modification (Axtell, 2013a; Borges and Martienssen, 2015). Small RNAs can be categorized into microRNAs or small interfering RNAs (siRNA) according to their biogenesis. MicroRNAs are derived from single-stranded hairpin RNA transcripts, which are usually transcribed from intergenic regions but can also originate from within protein-coding genes (Voinnet, 2009). After transcript processing, the mature microRNAs silence the expression of target genes by complementary binding to their RNA transcripts, inducing mRNA cleavage or blocking mRNA translation (Llave, 2002). The other category of small RNAs, siRNAs, can be further classified as heterochromatic siRNAs (hc-siRNAs), secondary siRNAs and natural antisense transcript siRNAs (NAT-siRNAs) (Axtell, 2013a). Hc-siRNAs are 23–24 nt long siRNAs, derived from repetitive regions that are involved in chromatin modifications (Matzke et al., 2009). Secondary siRNAs are generated from microRNA-guided cleavage, and are also referred to as phased siRNA (phasiRNA). phasiRNA are derived from both protein-coding or non-coding transcripts and can function in *cis* or *trans* (*trans*-acting siRNA, ta-siRNA) to negatively regulate gene expression (Vazquez et al., 2004; Montgomery et al., 2008). In monocots such as rice and maize, miR2118 triggers 21-nt phasiRNAs production, whereas miR2275 triggers a class of 24-nt phasiRNAs, which was originally thought to be missing in eudicots (Johnson et al., 2009; Song et al., 2012; Jeong et al., 2013). A recent study reported that miR2275 and the 24-nt phasiRNA pathway is widely present in eudicots and is specifically enriched in meiosis during pollen development (Xia et al., 2019).

Long non-coding RNAs (lncRNAs) are transcripts larger than 200 nt that do not encode proteins. Similar to mRNA, most lncRNAs have 3′ poly A tails and 5′ caps (Chekanova, 2015). There are also many non-polyadenylated lncRNAs mainly characterized from Arabidopsis including the well-studied lncRNAs *COLDAIR* and *COLDWRAP* (Heo and Sung, 2011; Di et al., 2014; Kim and Sung, 2017). The three Arabidopsis lncRNAs, *COOLAIR*, *COLDAIR* and *COLDWRAP* mediate flowering time by epigenetically suppressing *FLOWER LOCUS C (FLC)* expression through recruiting polycomb repressive complex 2 (PRC2) to *FLC.* PRC2 promotes histone H3K27 methylation which represses *FLC* expression (Liu et al., 2010; Heo and Sung, 2011; Kim and Sung, 2017). LncRNAs are also found to be involved in abiotic stress in plants. A cold response lncRNA *SVALKA* (*SVK*) transcribed from C-repeat binding factor (*CBF*) inhibits the expression of *CBF1* which is important to *Arabidopsis* cold tolerance and apple bud dormancy (Kindgren et al., 2018; Artlip et al., 2019).

The molecular control of the dormancy cycle in fruit species has mainly focused on the regulation of *DORMANCY-ASSOCIATED MADS-box* (*DAM*) genes (da Silveira Falavigna et al., 2018). The partial deletion of six tandemly repeated *DAM* genes was found to be associated with the peach (*Prunus persica*) *evergrowing* (*evg*) mutant that fails to enter dormancy (Bielenberg et al., 2008). Phylogenetic analysis demonstrated that *DAM*s are related to Arabidopsis *SHORT VEGETATIVE PHASE* (*SVP*) and SVP-like (*SVL*) protein from poplar (da Silveira Falavigna et al., 2018). Epigenetic regulation of *SVL* and MADS-box genes during dormancy was reported in poplar and *Prunus* spp. (Leida et al., 2012; Saito et al., 2015; Anh Tuan et al., 2016; Conde et al., 2017; Guo et al., 2017; Rothkegel et al., 2017; Singh et al., 2018). Studies from several genome-wide analyses of microRNAs and siRNAs suggested microRNAs were involved in chilling induced dormancy release in poplar and pear (Ding et al., 2014; Bai et al., 2016; Niu et al., 2016). Pear-specific miR6390 was found to suppress pear *DAM1* expression during endodormancy release in Chinese white pear (Niu et al., 2016) but this microRNA was not identified in Japanese pear (Bai et al., 2016). Poplar miR160 which targets the auxin pathway was significantly upregulated during dormancy release induced by chilling (Ding et al., 2014). Barakat et al. (2012) also identified microRNAs that respond to cold stress in peach vegetative buds (Barakat et al., 2012). However, the molecular function of non-coding RNAs during dormancy release has not been analyzed yet in peach flower buds.

In this study, we profiled genome-wide mRNA, long noncoding RNA, and small RNA over the course of endodormancy to bud break in peach. This data was originally examined at the DAM locus (Zhu et al., 2020), revealing that small RNAs localized in the DAM locus were induced in response to warm temperatures. Here we use that same data but expand the analysis to the whole genome. The co-expression patterns of small RNAs and lncRNAs revealed the potential non-coding RNAs responsive to ecodormancy or bud break. Our interaction networks of microRNAs and lncRNAs provide the candidate non-coding RNAs involved in endo-to eco-dormancy transition and dormancy release for future investigation.

## Materials and Methods

### RNASeq and Small RNASeq Datasets

We obtained the strand specific total RNA sequencing and small RNA sequencing data originally reported in Zhu et al. (2020), in which the data was analyzed only for reads mapping to the DAM locus. The full set of reads is available from NCBI Bioproject PRJNA493230 with accession numbers indicated in Supplementary Table S1. Briefly, cut shoots with fully dormant floral buds from the peach cultivar “John Boy” were placed in chilling conditions (4°C). RNA was extracted from dormant floral buds after 0 (T1), 500 (T2), and 1000 CH (T3). The branches were moved to (20°C) to induce bud break and further RNA collections were obtained from emerging flowers after day 3 (D3) and day 7 (D7) of warm exposure. Three biological replicates were sampled for RNA extraction at each time point, individually barcoded during library construction for both small RNA and strand specific total RNA, and sequenced on an Illumina Hi-Seq.

### Small RNA Annotation and Classification

Each time point has three biological replicates for a total of 284 million reads ranging from 14.7 to 23.6million reads per library. Raw reads were trimmed by cutadapt (Martin, 2011) to remove sequencing adaptors and reads not in the range of 18–30 nt. To remove rRNA contamination, reads aligning to rRNA small or large subunits from SILVA (Quast et al., 2013) were filtered out. The clean libraries were then processed by ShortStack (Axtell, 2013b; Johnson et al., 2016) to annotate the small RNA loci using the default setting with uniquely-weighting mode (Johnson et al., 2016). First, 284,934,616 reads from fifteen libraries were merged to identify small RNA clusters across the genome. Next, the major RNA (i.e. most abundant RNA at a locus) for each small RNA clusters was compared by BLAST with the parameters blastn-short task and an e-value cutoff of 0.1 to *Prunus persica* tRNA, rRNA, snRNA, microRNA sequences from Rfam 13.0 (Kalvari et al., 2018) and ta-siRNA sequences from tasiRNAdb (Zhang et al., 2014). The hc-siRNA loci were identified as meeting all of the following criteria: 1) the small RNA locus overlaps with repetitive region annotation from Phytozome 13 (https://phytozome-next.jgi.doe.gov/info/Ppersica_v2_1); 2) the major RNA sequence from the small RNA locus does not match known rRNA, tRNA, snRNA, microRNA and ta-siRNA sequences; 3) the major RNA sequence from the small RNA locus is in the range of 23–24 nt.

After removing rRNA reads and reads with read length outside the range of 18 to 30-nt, 207.7 million clean reads were aligned to the peach reference genome Prunus persica v2.0 (Verde et al., 2017). One library from 1000 CH was identified as an outlier with an 8.8% mapping rate, while the average mapping rate across other libraries was 82% (Supplementary Table S1). The size distribution showed the majority of the reads in the high quality libraries are either 21 or 24-nt, while the outlier library had a large proportion of 22-nt and 23-nt reads (Supplementary Figure S1). We decided to discard this library from further analysis, thus, the 1000 CH time point included two replicates instead of three in the downstream analyses.

Novel microRNA loci were predicted using mirDeep2 (Friedländer et al., 2012) with the following inputs: clean reads from the fourteen libraries, 180 known *Prunus persica* microRNA sequences, and 453 microRNA from two other Rosaceae species, *Malus domestica* and *Fragaria vesca,* from miRBase (Kozomara et al., 2019). The predicted novel microRNAs matching the known microRNA from peach and the two Rosaceae species were removed. The remaining 21–22-nt microRNAs located in the non-coding regions, with a significant randfold *p*-value (<0.05) and a mirDeep score greater than 3 were regarded as true novel microRNAs. MicroRNA structural sequences were predicted by RNAfold contained in the ViennaRNA v1.8.4 Package (Lorenz et al., 2011). Novel microRNAs were assigned a temporary name, and will be submitted to miRBase for final name assignment.

### MicroRNA and siRNA Quantification

The read counts for siRNA cluster loci were calculated by HTSeq-count (Anders et al., 2015). Known and novel microRNA expression levels were counted by mirDeep2 quantifier (Friedländer et al., 2012).

### Gene Expression Quantification

A total of 342,919,300 strand-specific paired-end Illumina reads from 15 libraries were analyzed and gene counts were obtained for each gene locus as previously described (Yu et al., 2020). Briefly, rRNA reads were removed and 96.41% of remaining reads were successfully mapped with STAR v2.5.3a (Dobin et al., 2013) to peach genome Prunus persica v2.0 (Verde et al., 2017). Reads per gene were counted with HTseq-count (Anders et al., 2015) and gene-level differential expression assessed with DESeq2 (Love et al., 2014).

### Long Non-coding RNA Identification and Quantification

To remove ribosomal RNA (rRNA) and transfer RNA (tRNA) contamination in the RNASeq libraries, bowtie2 (Langmead and Salzberg, 2012) with default settings was used to filter out reads aligning to rRNA small and large subunits sequences downloaded from SILVA (Quast et al., 2013) or to tRNA sequences predicted by tRNAscan-SE 2.0 (Chan and Lowe, 2019). Remaining reads were then aligned to *Prunus persica* v2.0 (“Lovell 2D” genotype) by STAR 2.5.3a (Dobin et al., 2013) and transcripts were assembled by Stringtie 2.0.6 (Pertea et al., 2015). The lncRNAs were predicted through the machine learning method FEELnc (Wucher et al., 2017) using 3,301 peach lncRNA sequences from GreeNC (Paytuví Gallart et al., 2016) as training data. The predicted lncRNAs were quantified by HTSeq-count (Anders et al., 2015) at the gene level.

### Differential Expression Analysis

Read counts from mRNA, lncRNA, microRNA or siRNA were normalized and analyzed by DESeq2 (Love et al., 2014). Sample variances were computed from the regularized log transformation of raw counts. Principal component analysis explained the largest and the second-largest variance among samples. The significantly differentially expressed mRNA, lncRNA, microRNAs and siRNAs were determined by pairwise comparisons for five time points with expression change of at least two-fold and an adjusted *p*-value smaller than 0.05 (Fold change >2 and FDR <0.05).

### Co-expression Analysis

Normalized counts from DESeq2 were analyzed by the R package “WGCNA” to identify the co-expression modules, following the package tutorials (Langfelder and Horvath, 2008). For hc-siRNA, a minimum of 300 loci were included in each module. For microRNA, the minimum module size was set to contain 10 microRNAs. For lncRNA, the minimum module size was set to 30.

### Identification of miRNA Targets

MicroRNAs target transcripts were identified from both publicly available degradome datasets and computational prediction by psRNATarget V2 (Dai et al., 2018). Two degradome sequencing datasets from the peach cultivar “Lovell” were obtained from NCBI SRR504537 (Zhu et al., 2012) and SRR948302 (Luo et al., 2013). Adaptors and low-quality reads were removed by skewer (Jiang et al., 2014). Clean degradome sequencing reads, mRNA transcript sequences from the peach transcriptome, and 250 microRNA sequences were analyzed by CleaveLand4 (Addo-Quaye et al., 2009) to identify microRNA target transcripts. The prediction of 24-nt phasiRNA triggered by miR2275 was performed by psRNATarget V2 (Dai et al., 2018) using ppe-miR2275 sequence and the siRNA clusters with major RNA of 24 nt. The target transcripts with degradome peak categories 0–2 were kept as true positive targets. psRNATarget V2 predicted microRNAs and lncRNA target sites with E-value less than 2 to reduce the false positive targets.

### Gene Networks Construction and Pathway Enrichment

The genes identified as DE microRNA targets were constructed into gene networks based on protein-protein interaction (PPI) networks from STRING v11 database (Szklarczyk et al., 2019). The degree of connectivity and hub score for each gene were calculated by R package “igraph” (Csardi et al., 2006). The genes with high degree and high hub score were identified as hub genes in the network. Gene ontology enrichment pathways were analyzed and built by AgriGO 2.0 (Tian et al., 2017). Kyoto Encyclopedia of Genes and Genomes (KEGG) pathway enrichment was analyzed by Wilcoxon rank sum test using the *p*-values calculated by DESeq2 under the background of the Arabidopsis genome in R package “KEGGREST” (Tenenbaum, 2016).

## Results

### Small RNA Expression Patterns are Similar From Endodormancy to Ecodormancy Then Change Significantly at Bud Break

Zhu et al. (2020) reported transcriptional patterns of non-coding and coding RNA originating from the DAM locus across five floral bud dormancy time points. We utilized the same small RNASeq and strand specific RNASeq data to examine genomewide patterns of transcription. Data was obtained from five time points during peach bud dormancy release, including two endodormant bud stages at 0 and 500 CH, an ecodormant bud stage at 1000 CH, as well as two bud break stages, day 3 and day 7 after being moved into conditions to induce flowering (Zhu et al., 2020). These time points are referred to as T1, T2, T3, D3, and D7, respectively.

To explore whether small RNAs are involved in dormancy release, we performed a *de novo* annotation of the small RNA loci via ShortStack (Axtell, 2013b). In total, we identified 72,984 putative small RNA loci and defined a single major RNA sequence for each locus, which were used for all downstream analysis. 3,972 loci were identified as peach tRNA, rRNA, snRNA, microRNA, or ta-siRNAs (Table 1). Of the remaining loci (69,012), the majority (66,561) were between 20 and 24 nt in length and were classified as siRNA.

**TABLE 1:** The summary of small RNA loci by major RNA size.

	20 nt	21 nt	22 nt	23 nt	24 nt	Other Length
rRNA	0	7	2	0	1,043	35
tRNA	6	36	4	7	605	288
snRNA	0	8	4	1	739	148
miRNA	4	58	10	1	935	23
ta-siRNA	0	4	0	0	4	0
siRNA	0	1,042	248	133	65,130	0
other	16	0	0	0	0	2,443
total	26	1,155	268	142	68,456	2,937

**FIGURE 1:**
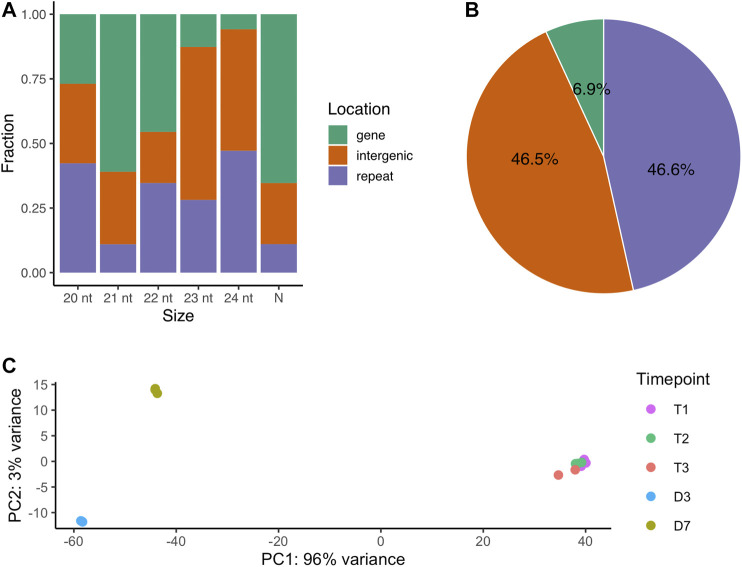
The genomic origins of small RNA loci **(A)** The fraction of all small RNA loci located across genomic regions by major RNA size. N represents major RNAs that are either shorter or longer than 20-24 nt **(B)** The percentage of novel siRNA loci by genomic regions **(C)** PCA analysis of small RNA loci expression over dormancy progression. T1, 0 CH; T2, 500 CH; T3, 1000 CH; D3, day 3 of warm conditions; D7, day 7 of warm conditions.

The localization of the 72,984 small RNA loci across the genome varied by length. The majority of 21-nt small RNAs are located in gene regions (61%). The 23-nt small RNA loci are mainly in the intergenic regions and 24-nt small RNA loci are mostly in the repeat regions (Figure 1A). Looking specifically at the 66,561 novel siRNA loci (Table 1), 45.6% loci were located in the repetitive element regions (repeat), while 46.5% loci were in the non-coding regions (intergenic) and only 6.9% were in the protein-coding regions (Figure 1B).

The expression levels of the small RNA loci were quantified and were subjected to principal component analysis (PCA). The first and second components of PCA explained 96% and 3% of the sample variances respectively, indicating a large difference in overall small RNA expression between dormancy (including endodormancy and ecodormancy) and budburst stages (Figure 1C). Our results suggest that the small RNA expression remained largely consistent in the dormant buds during endodormancy and ecodormancy as little overall expression variation was observed between these two stages. In contrast, variation can be observed between the D3 and D7 stages, indicating siRNA expression differed between the two flowering days.

### MicroRNAs are Differentially Expressed During Dormancy Release

Peach microRNAs have been identified across tissues (Zhu et al., 2012; Luo et al., 2013), during fruit development (Zhang et al., 2016; Shi et al., 2017), and in response to chill (Barakat et al., 2012). We specifically focused on the microRNA response during peach bud dormancy transition and release. We identified 250 microRNAs, including 88 known peach miRNAs in miRBase and 162 novel miRNA. The expression level of each microRNA was quantified and their relative expression within each time point was calculated. The expression of 15 microRNAs accounted for over 90% of the expression of all microRNAs (Supplementary Figure S2). In the endodormancy and ecodormancy stages (T1, T2, and T3), two microRNAs (miR167b and miR167c) constituted a high fraction of overall reads, but were a much lower fraction in the bud burst stages (D3 and D7).

**FIGURE 2:**
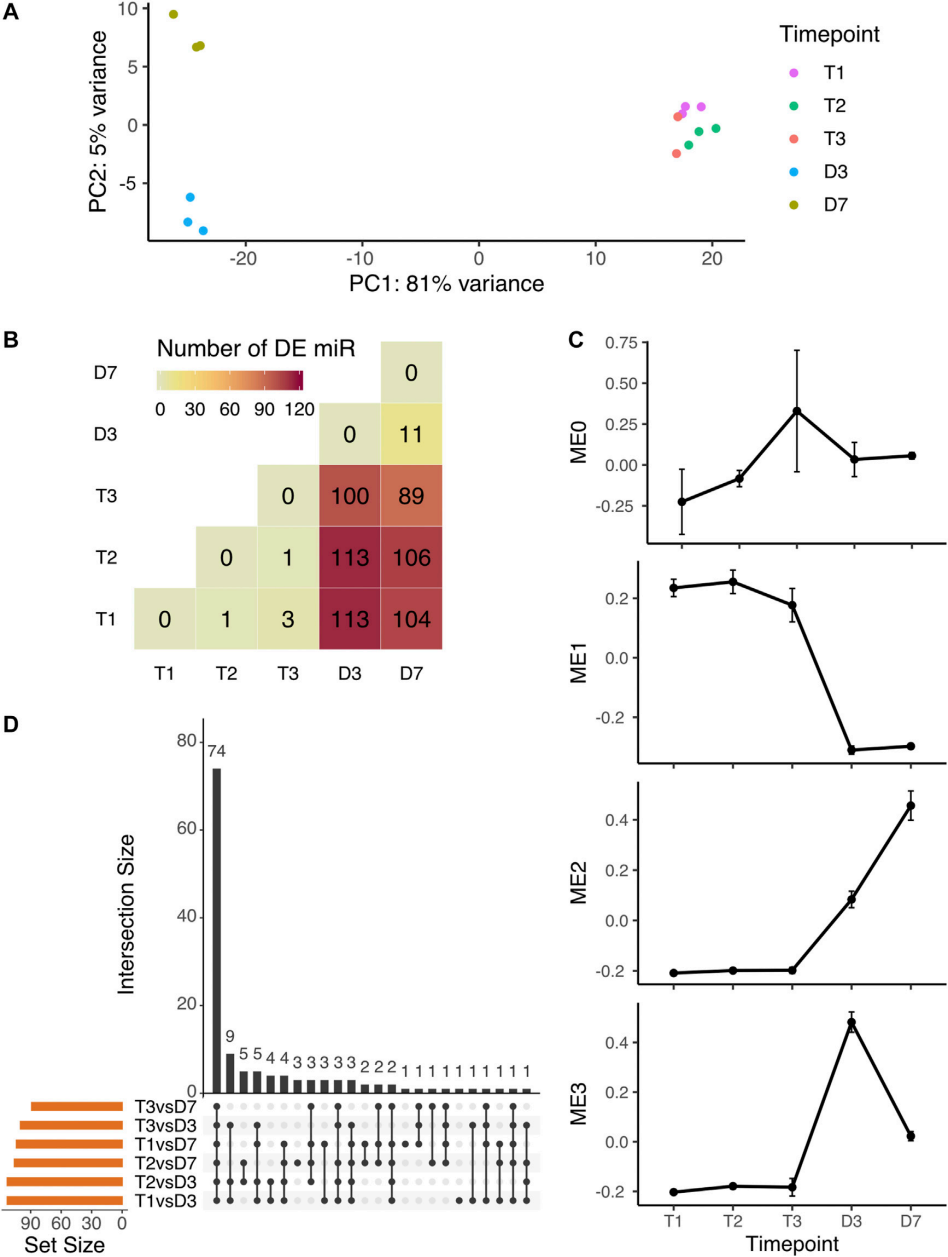
Expression variation of microRNA during peach bud dormancy release **(A)** PCA analysis of microRNA expression over five time points in dormancy **(B)** The pairwise comparison indicates the number of differentially expressed microRNA detected by all statistical tests (all-by-all comparison of five time points). The majority of detected DE miRNAs were from the six comparisons with a dormant stage compared to a bud break stage **(C)** Specifically for the tests comparing dormancy (T1, T2, and T3) to bud burst (D3 and D7) time points, the same miRNAs were often identified in multiple tests. The miRNAs shared across different combinations of statistical tests are indicated by the black dots (tests) and black bars (number of shared miRNAs) **(D)** The expression profiles of the module eigengene (mean ± SE), i.e. the overall expression pattern representing the genes in the module, from each microRNA co-expression module identified by WGCNA.

The PCA analysis of microRNA expression showed the same clustering pattern as overall small RNA expression. The endodormant and ecodormant stages clustered together with the largest PCA component (81%) clearly differentiating the dormant and bud burst stages. The plot also shows separation (5%) between the two bud burst stages (Figure 2A). To identify the individual microRNAs changing in expression during dormancy phase transition and dormancy release, we performed pairwise comparisons among the five time points. More than 100 microRNA were differentially expressed (DE) between dormant buds and flowering buds (Figure 2B). 74 out of 132 microRNAs were significant in all six comparisons between dormant stages and bud-burst stages (T1 vs D3, T1 vs D7, T2 vs D3, T2 vs D7, T3 vs D3 and T3 vs D7) (Figure 2C).

Co-expression clustering among all microRNAs over the five time points grouped microRNAs into four modules (MEs) (Supplementary Figure S3A), each with a unique expression profile (Figure 2D). Of 250 microRNAs, 155 had high expression levels at the dormant stages and were downregulated after bud-burst (Supplementary Figure S3B). Over 66% of the differentially expressed microRNAs between dormant and bud-burst stages were clustered into ME1 (Supplementary Table S2). ME2 and ME3 displayed a pattern of low expression levels at endodormancy and ecodormancy stages, then were upregulated at bud-burst (Figure 2D). All DE microRNAs not found in ME1 were found in ME2 or ME3. The 13 microRNAs in ME0 showed a general pattern of upregulation at ecodormancy, however, none of them were statistically significant in the DE analysis (Supplementary Table S2). Overall, our primary result from both DE analysis and coexpression analysis is that a large number of microRNAs are significantly downregulated after bud break.

### Genes Targeted by microRNAs are Involved in Stress Response Pathways During Dormancy Release

Two degradome (also known as parallel analysis of RNA ends or PARE) sequencing libraries from peach genotype “Lovell” (Zhu et al., 2012; Luo et al., 2013) are publicly available. The libraries were generated from pooled leaf, fruit, and dormant bud tissues collected at T1, T2, T3 and D3 stages, mirroring the same experimental design as our samples. To determine the transcripts that microRNAs may regulate, we identified 211 microRNA target genes predicted computationally and by the public degradome data. Of the 211 degradome-supported target genes, 70 genes are targets of 35 known microRNAs and 141 genes are targets of 52 novel microRNAs (Supplementary Table S3).

**FIGURE 3:**
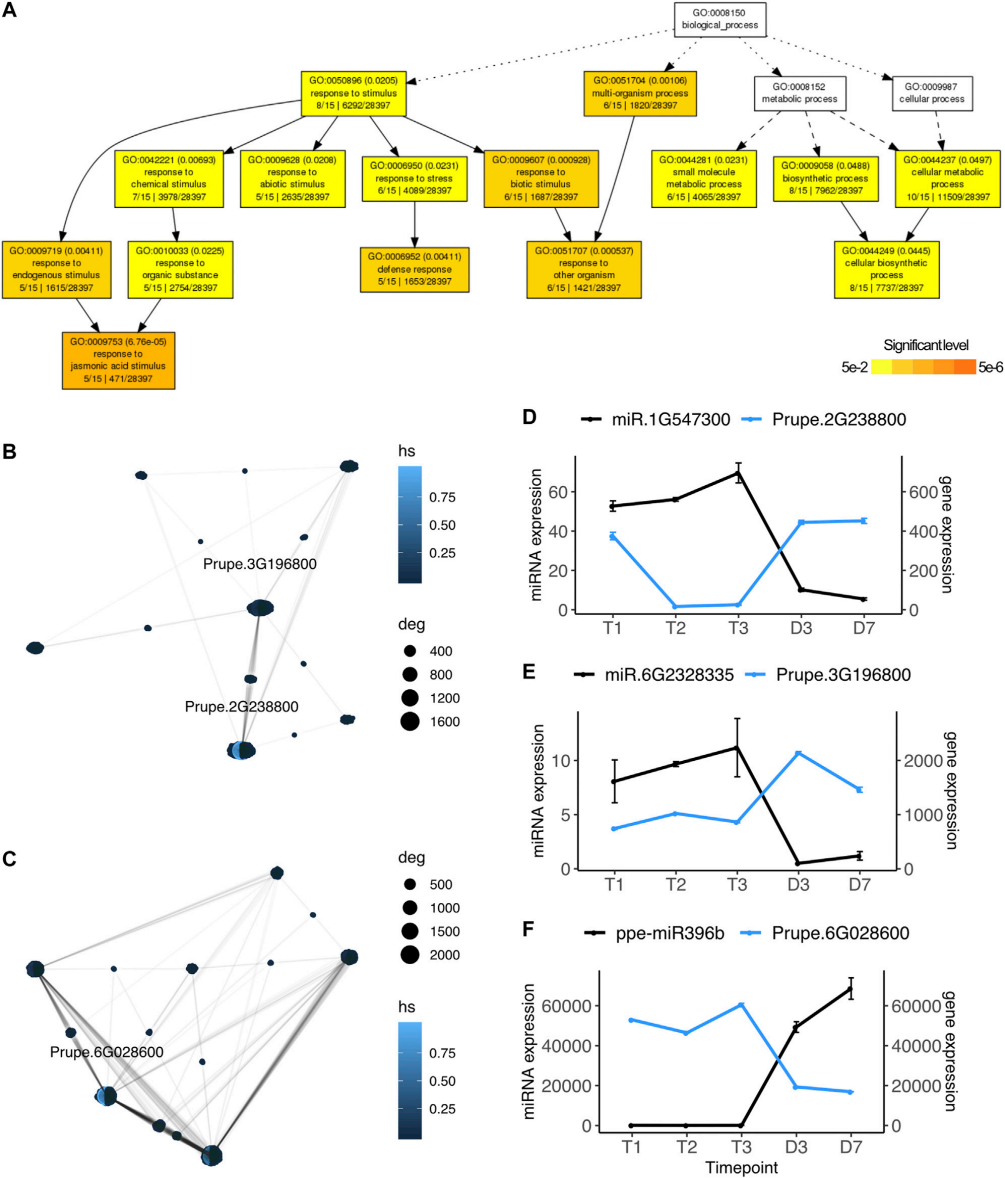
microRNA regulated pathways during dormancy release **(A)** Gene ontology network of microRNA targeted genes that displayed opposite expression profiles. GO terms in white indicated no significance **(B)** Gene interaction network of upregulated genes targeted by downregulated microRNA **(C)** Gene interaction network of downregulated genes targeted by upregulated microRNA **(D)**–**(F)** Gene expression profiles (mean ± SE) of microRNAs (in black) and their targeted hub genes (in blue) identified by the networks **(B)** and **(C)**. Prupe.2G238800, Rubisco activase; Prupe.3G196800, acetoacetyl-CoA thiolase; Prupe.6G028600, heat shock protein 90.

Using the strand-specific paired-end Illumina RNASeq reads also publicly available for this experiment, we performed the same pairwise differential expression analysis on the 26,872 peach protein-coding genes and identified significantly differentially expressed genes ranging from 334 to 4,619 in each comparison (Supplementary Table S2). Among the DE genes, only 44 genes were also identified as known or novel microRNA targets. They were differentially expressed between dormant stages and bud-burst stages, mainly identified in the comparisons of T1 vs D3 and T2 vs D7 (Supplementary Table S2). To explore potential genes and pathways targeted by the DE microRNAs, we divided the target genes into four groups: upregulated genes targeted by downregulated microRNAs, upregulated genes targeted by upregulated microRNAs, downregulated genes targeted by upregulated microRNAs, and downregulated genes targeted by downregulated microRNAs. Target genes having opposite expression patterns with their paired microRNAs at specific time points may indicate miRNA regulation has an important role in controlling transcription at those particular time points. Among the 44 significant DE genes, 17 genes have opposite expression profiles as their paired microRNAs (upregulated genes targeted by downregulated microRNAs or downregulated genes targeted by upregulated microRNAs). Gene ontology enrichment analysis indicated these 17 genes are involved in the biological pathways including response to jasmonic acid stimulus (GO:0009753, FDR = 6.76e-0.5), defense response (GO:0006952, FDR = 0.004), abiotic stress (GO:0009628, FDR = 0.231) and cellular metabolic process (GO:0044237, FDR = 0.0497) (Figure 3A).

From those 17 genes with expression patterns opposite their paired microRNAs, we built gene interaction networks retrieved from the protein-protein interaction database STRING v11 (Szklarczyk et al., 2019). Hub genes were identified based on highest degrees of connectivity and hub scores. Two hub genes, Purpe.2G238800 (degree = 1,640, hub score = 1.0) and Prupe.3G196800 (degree = 1,194, hub score = 0.15) were identified from the upregulated gene network (Figure 3B). The expression of novel microRNA miR.1G547300 was downregulated at D3, while its target gene Prupe.2G238800 was upregulated at D3 (Figure 3D). Prupe.2G23880 encodes rubisco activase, an ortholog of Arabidopsis *RCA* (AT2G39730), which is a chloroplast heat-labile enzyme regulating the activity of the photosynthesis key protein Rubisco (Salvucci and Ogren, 1996). The expression profiles of Prupe.2G238800 suggested that the photosynthesis pathway may be inhibited by the microRNA miR.1G547300 during endodormancy and ecodormancy and reactivated at bud break when the miR.1G547300 expression drops. Another gene identified as a hub gene in this network is Prupe.3G196800, an acetoacetyl-CoA thiolase (thiolase II), which was also significantly upregulated at D3 opposite to its paired microRNA miR.6G2328335 (Figure 3E). Thiolase II is an enzyme that negatively regulates isoprenoid production and is involved in antioxidant defense to adapt to salt stress (Soto et al., 2011). Prupe.6G028600 was identified as a hub gene (degree = 2,160, hub score = 1.0) in the gene network of downregulated genes targeted by upregulated microRNAs (Figure 3C). Prupe.6G028600 was downregulated at D3 and it is targeted by miR396b which was upregulated at bud break. MiR396b was also one of the microRNAs contributing a large fraction of overall miRNA expression at D3 and D7 (Supplementary Figure S2). The higher expression level of miR396 in flowers than in dormant flower buds is in contrast to the previous finding that miR396 was highly expressed in dormant vegetative buds (Barakat et al., 2012). miR396b targets gene Prupe.6G028600, an ortholog of Arabidopsis heat shock protein 90 (Hsp90, AT5G56000). A recent study reported that Hsp90 acts as a microRNA loading chaperone that plays an important role in transporting AGO1-microRNA during microRNA silencing (Bologna et al., 2018).

A number of microRNAs had expression changes in the same direction as their target genes, i.e., both up-regulated or both down-regulated. This may indicate other regulatory mechanisms are altering the expression of these genes in concert or in opposition to the miRNA expression. For the 28 genes in this category, they were enriched in response to chemical stimulus (GO:0042221, FDR = 0.00176), response to abiotic stress (GO:0009628, FDR = 0.00308) and alcohol metabolic process (GO:0006066, FDR = 0.00345) (Supplementary Figure S4A). Gene interaction network analysis identified a hub gene, Prupe.4G138900, upregulated at bud break, and a hub gene, Prupe.1G173900, downregulated at bud break. Both genes and their paired microRNAs showed similar expression profiles across the experimental time points (Supplementary Figure S4B,C). Prupe.4G138900 is an ortholog of Arabidopsis cold stress gene *LOS1* (a translation elongation factor 2-like gene), which is upregulated under cold conditions and is a key signaling component for freezing tolerance (Guo et al., 2002). However, the vernalization and flowering time were not affected in the *los1-1* mutant (Guo et al., 2002). Prupe.1G173900 encodes a ribosomal protein L10 (RPL10) which consists of 60S large subunit ribosomes (Dick and Trumpower, 1998). The RPL family was reported previously to be involved in translation and protein synthesis during plant development and stress (Byrne, 2009; Falcone Ferreyra et al., 2013). Based on the GO term enrichment analysis and the specific hub genes, genes positively correlated in expression with microRNAs relate to cold stress response and developmental stage transitions.

### Peach miR6285 and miR2275 are Differentially Expressed Between Endodormancy to Ecodormancy

**FIGURE 4:**
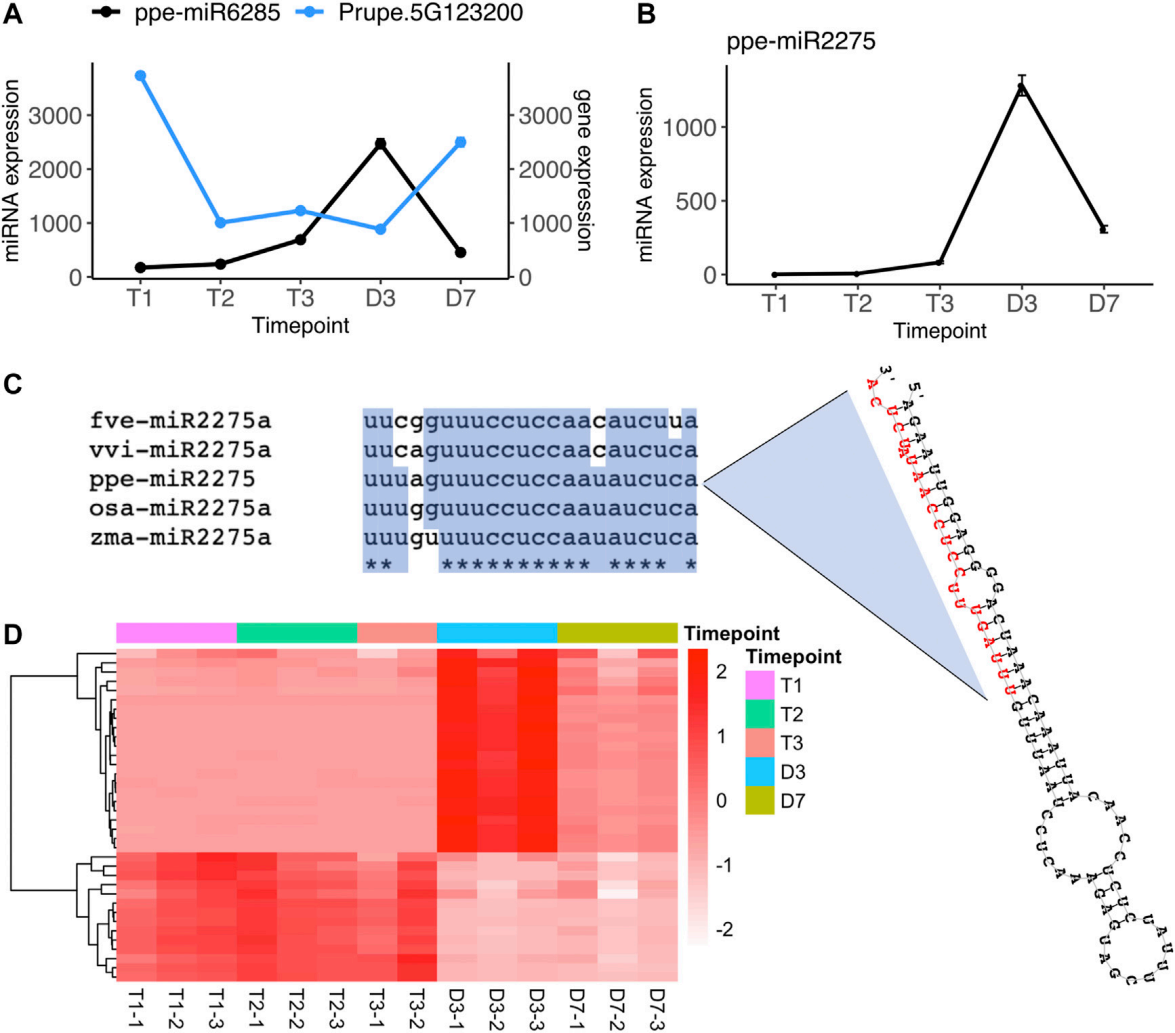
Two microRNAs, ppe-miR6285 and ppe-miR2275 are significantly upregulated at ecodormancy **(A)** Gene expression profile of ppe-miR6285 and its target gene Prupe.5G123200 (mean ± SE) **(B)** Gene expression profile of ppe-miR2275 (mean ± SE) **(C)** ppe-miR2275 pre-microRNA structure predicted by RNAfold (Lorenz et al., 2011), with mature miR2275 sequence in red, and sequence alignment of mature miR2275 in peach (ppe), rice (osa), maize (zma), strawberry (fve) and grape (vvi) **(D)** The expression of predicted 24 nt-phasiRNA loci targeted by ppe-miR2275 with each row indicating a 24 nt-siRNA locus and with each column representing one sample. The color scale indicates relative expression levels of the 24 nt-siRNAs over the five time points.

In the comparisons of endodormancy to ecodormancy stages, we identified three microRNAs (ppe-miR3627-5p, ppe-miR6285, and miR.3G1416188) significantly differentially regulated between T1 vs. T3 and one microRNA (ppe-miR6285) differentially expressed between T2 vs. T3 (Figure 2B, Supplementary Table S2). Only miR6285 has a predicted target from the degradome sequence analysis, Prupe.5G123200. This gene was found to be significantly downregulated at T3 while ppe-miR6285 was upregulated at T3 (Figure 4A, Supplementary Table S4). Prupe.5G123200 encodes a asparagine-rich protein (NRP) with a development and cell death (DCD) domain. An NRP was recently reported to be involved in the regulation of ABA signaling in Arabidopsis (Zhu et al., 2018). The *nrp* mutants in Arabidopsis and soybean plants are hypersensitive to salt and osmotic stress (Hoepflinger et al., 2011; Reis et al., 2016) and Arabidopsis overexpressing NRP exhibited high sensitivity to ABA treatment (Zhu et al., 2018). Stress response and ABA signaling related genes are known to be involved in peach endodormancy to ecodormancy transition (Yu et al., 2020). The differentially expressed miR6285 and *NRP* gene between T1 and T3 not only further supports that stress response genes play a critical roles during dormancy phase transition, but also provides additional evidence that ABA and stress response pathways are likely regulated by microRNAs during endo-to eco-dormancy transition.

Another differentially expressed microRNA, miR.3G1416188 is not included in miRBase although its sequence has been identified in previous studies (Barakat et al., 2012; Luo et al., 2013). However, neither of the studies have further investigated its function. We found this 22-nt microRNA has 95% similarity to miR2275 in rice (*Oryza sativa*), which is specifically enriched in anthers and triggers 24-nt phasiRNA production from transcripts originating at *PHAS* loci (Fei et al., 2016). This miR2275-driven 24-nt phasiRNA pathway has not only been identified in monocots but also has been recently found enriched in meiotic pollens in eudicots such as strawberry and litchi (Xia et al., 2019). The microRNA miR.3G1416188 (also named as ppe-miR2275) identified in our study has a stem-loop precursor and mature microRNA sequence orthologous to miR2275 in strawberry, grape, rice, and maize (Figure 4C). From the overall set of siRNA clusters, we found that 36 loci were targeted by ppe-miR2275 and had a phase score greater than 30, indicating these as *PHAS* loci in the peach genome. The expression level of ppe-miR2275 was upregulated at T3 and then peaked at D3 (Figure 4B). 22 out of 36 predicted phasiRNA loci also peaked at D3, similar to the expression pattern of ppe-miR2275 (Figure 4D). The expression profiles of ppe-miR2275 and its target 24-nt phasiRNA loci suggested that the miR2275/24 nt-phasiRNA pathway is likely initiated at ecodormancy and enriched in meiosis during peach bud burst, likely to support pollen development.

### Heterochromatic siRNA in Response to Bud Break

**FIGURE 5:**
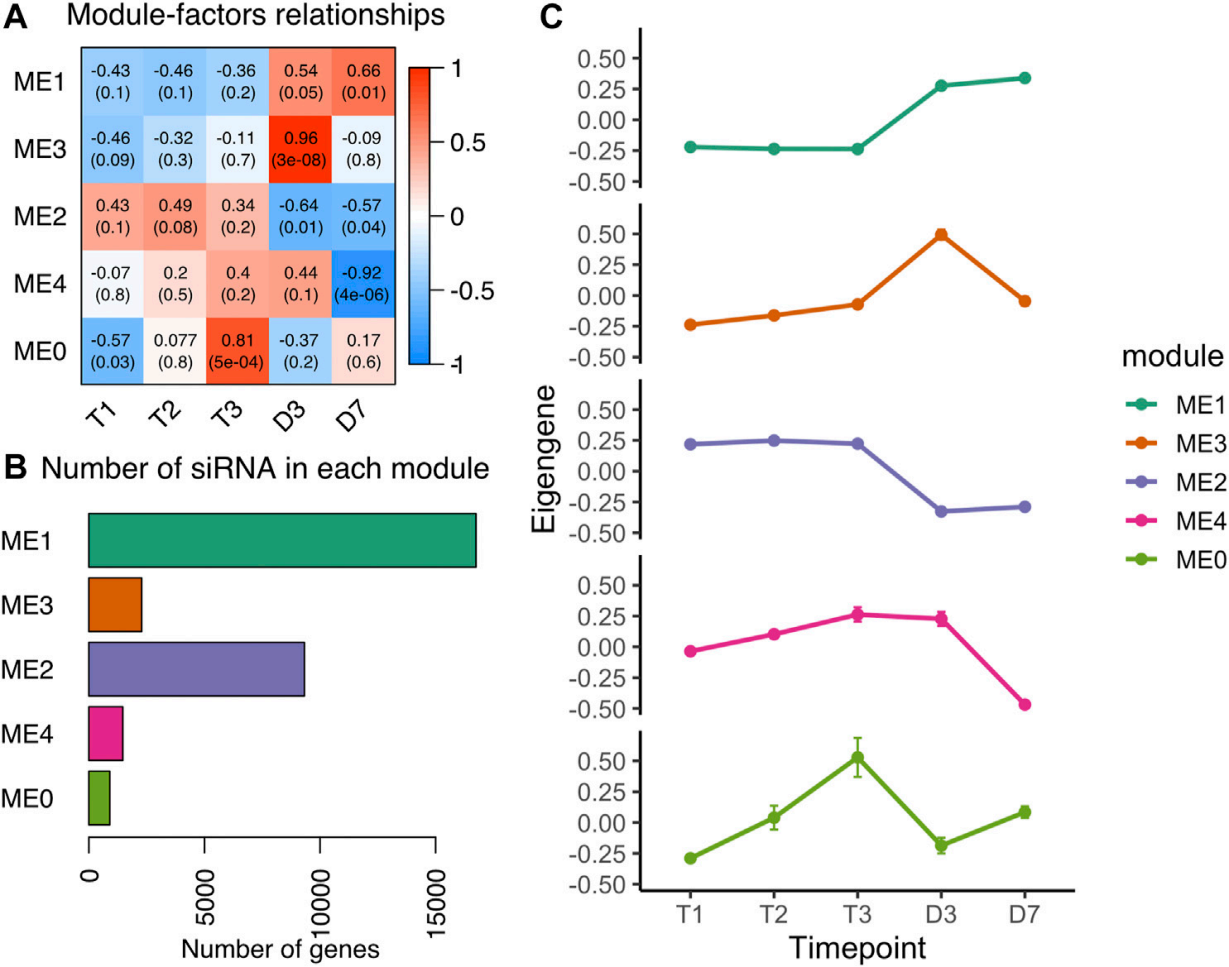
Co-expression analysis of hc-siRNA from dormancy to bud break **(A)** Module-trait relationships between modules to time points. A higher correlation score indicates a higher expression level at that time point **(B)** The number of hc-siRNAs in each module **(C)** The expression patterns of eigengene from the five modules (mean ± SE).

Heterochromatic siRNA (hc-siRNA) are generated from repetitive regions in plant genomes (Axtell, 2013a). The process of RNA directed DNA methylation (RdDM) is primarily regulated by the siRNAs originated from transposons and intergenic regions (Elvira-Matelot et al., 2016). Of 32,898 siRNA (46%) clusters located in the repetitive element regions (Figure 1B), we classified 32,343 siRNA clusters with 23–24 nt length as heterochromatic siRNAs. To investigate whether the hc-siRNA expression pattern changed during dormancy, we built a coexpression network that clustered 30,727 hc-siRNAs into five modules ranging from 904 to 16,747 hc-siRNAs in each module (Figures 5A,B). More than half of the hc-siRNAs clustered into ME1 with a common expression pattern (Figure 5B), specifically low levels of expression during chill in endodormancy and ecodormancy, and increased the expression levels in warm temperatures at bud break (Figure 5C). ME2 included 9,323 hc-siRNAs, with the members expressed highly in chilling conditions and downregulated in warm conditions (Figure 5C). The expression profiles of hc-siRNAs in our results suggested that major hc-siRNAs expression changes occur during the transition to warm temperatures and bud break.

### Long Non-Coding RNAs Have Distinct Expression Patterns Throughout Dormancy and Flowering Time Points

Long non-coding RNAs are another type of regulatory non-coding RNA previously profiled in the regulation of flowering time and stress responses (Di et al., 2014; Berry and Dean, 2015; Kim and Sung, 2017). We identified over 5,000 novel transcripts from our strand-specific RNASeq data. From these, 785 putative loci were predicted to generate 1,329 unique long non-coding transcripts. Based on the transcription direction of the lncRNAs in relationship to the closest mRNA annotated gene, they were categorized as either sense or antisense. The majority of the lncRNAs are antisense and 316 antisense transcripts overlap exons of annotated protein coding genes (Table 2).

**TABLE 2:** The classes of 1,329 long non-coding RNAs.

Direction	sense				antisense			
Type	genic		intergenic		genic		intergenic	
Location	intronic	exonic	upstream	downstream	intronic	exonic	upstream	downstream
LncRNA transcript #	66	0	226	235	26	316	237	223

**FIGURE 6:**
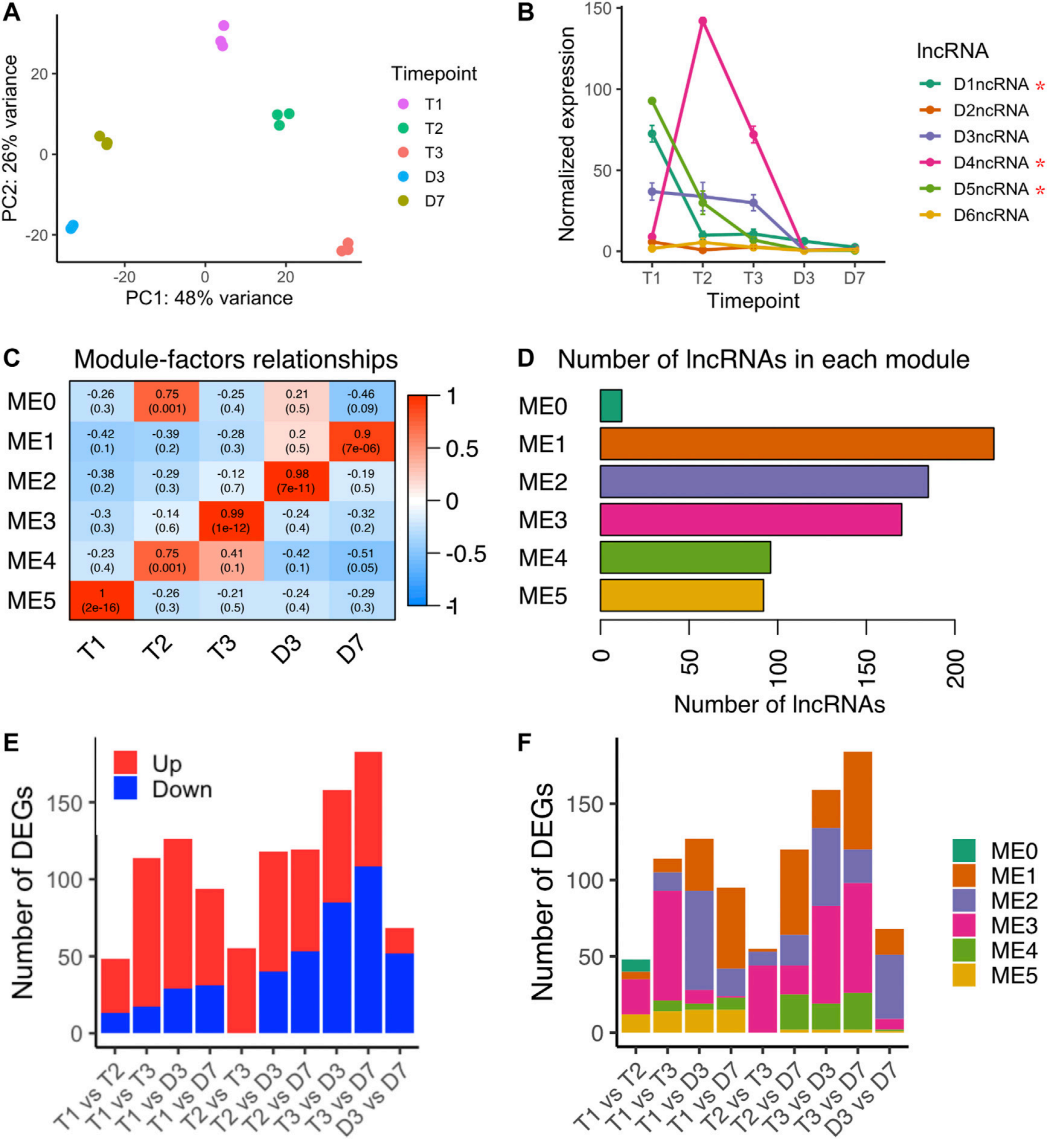
lncRNA expression variances during bud dormancy release **(A)** PCA analysis of lncRNA transcriptome variances over five time points **(B)** Expression profiles of six intronic non-coding RNAs in six *DAM* genes (mean ± SE). Red stars indicated the lncRNAs were differentially expressed between endodormancy and ecodormancy **(C)** Module-trait relationships between co-expression modules to time points **(D)** The number of lncRNA genes included in each co-expression module **(E)** The number of significantly upregulated lncRNA genes (red) and downregulated lncRNA genes (blue) in the pairwise comparison between time points **(F)** The number of differentially expressed lncRNA genes in each co-expression module.

Zhu et al. (2020) identified non-coding RNAs in the intron of *DAM3*, *DAM4*, and *DAM5* (Zhu et al., 2020)*,* however, our computational pipeline initially did not predict the same intronic ncRNAs. By manually annotating the *DAM* intronic ncRNAs using the updated DAM gene structure annotation provided from Zhu et al., (2020), we found intronic ncRNAs in the second intron of all six *DAM* genes, named as D1ncRNA to D6ncRNA corresponding to *DAM1* to *DAM6* (Supplementary Figure S5). D3ncRNA to D5ncRNA are the same intronic ncRNAs identified in Zhu et al., (2020), and D1ncRNA, D2ncRNA, and D6ncRNA were newly identified in this study. The six intronic ncRNAs in the *DAM* genes showed three distinct patterns: D2ncRNA and D6ncRNA had no obvious changes over time; D1ncRNA, D3ncRNA and D5ncRNA were downregulated as dormancy progresses; and D4ncRNA peaked at T2 (Figure 6B). The pattern suggests D4ncRNA was induced by chill in a similar expression pattern as Arabidopsis *COLDAIR* in vernalization (Heo and Sung, 2011). The upregulation of D4ncRNA during chill accumulation suggests D4ncRNA may be involved in inhibition of *DAM4* expression during peach bud dormancy. We also found three *DAM* ncRNAs (D1ncRNA, D4ncRNA, and D5ncRNA) that were significantly differentially expressed between endodormancy to ecodormancy (Figure 6B), suggesting that these three ncRNAs may be involved in dormancy regulation.

Including the *DAM* intronic ncRNAs, we quantified a total of 791 loci producing lncRNAs. A PCA analysis showed that the lncRNA transcriptomes separated by time point with the first PC explaining 48% of the variance and the second PC explaining 26% of the variance (Figure 6A). To investigate the lncRNAs sharing the same expression patterns, we performed weighted gene co-expression network analysis (WGCNA). Six modules ranging from 12 to 222 genes were identified (Figures 6C,D). Module-trait correlation analysis indicated the relationship between the lncRNAs in each module to the five time points. Higher correlation scores represent higher expression levels of the eigengene at the corresponding time point. The 92 lncRNAs in module 5 (ME2) peaked in expression level at T1, and decreased upon dormancy release (Figure 6D and Supplementary Figure S6). In contrast, module 3 (ME3) containing 170 lncRNAs peaked at ecodormancy stage (T3) and had low expression levels at other time points (Figure 6D and Supplementary Figure S6).

### LncRNA Were Differentially Expressed Between Endodormancy and Ecodormancy

Applying the same pairwise differential expression analysis reported for microRNAs, we identified differentially expressed lncRNAs in each time point comparison, ranging from 48 to 184 genes (Figure 6E). We found 114 differentially expressed lncRNAs between endodormancy (T1) to ecodormancy (T3). The majority of them were upregulated and clustered into ME3, suggesting that these lncRNAs are likely induced at ecodormancy (Figures 6E,F and Supplementary Table S5). 159 lncRNAs were differentially expressed between ecodormancy (T3) and budburst (D3), and more than half of the DE lncRNAs were downregulated at D3 (Figure 6E). Of these 159 DE lncRNAs, 64 and 51 were clustered into ME3 and ME2, respectively (Figure 6F and Supplementary Table S5).

lncRNAs often associate with and regulate nearby genes in the case of intergenic lncRNA and their corresponding overlapping gene in the case of genic lncRNA (Wucher et al., 2017). Intergenic lncRNA can act as a *cis*-acting element to regulate the nearby genes (Vance and Ponting, 2014). We identified partner genes as putative regulatory targets for the peach lncRNAs, yielding 408 genes that overlapped with the genic lncRNAs and 921 genes within 100 kb to intergenic lncRNAs. Of the 117 putative partner genes to the differentially expressed lncRNAs, only 27 genes were also differentially expressed between T1 and T3 including three *DAM* genes (*DAM1*, *DAM4* and *DAM5*) and their intronic ncRNAs (Supplementary Table S6). We further identified 48 DE lncRNAs between T1 and T2 (Supplementary Table S7), 55 DE lncRNAs between T2 and T3 (Supplementary Table S8), and 158 DE lncRNAs between T3 and D3 (Supplementary Table S9). We used the partner transcripts of the lncRNAs differentially expressed between T1 and T2, T2 and T3, and T3 and D3 to identify enriched metabolic pathways in response to chill, endo-to eco-dormancy transition and warm. Pathway enrichment analysis was performed using the annotation from KEGG database (Kanehisa and Goto, 2000; Kanehisa et al., 2019). Genes co-localized with DE lncRNAs from T1 vs T2 were enriched in seven pathways, but none of them were significant (Supplementary Figure S7A). Gene co-localized with lncRNAs differentially expressed between endodormancy and ecodornancy were significantly enriched in the flavonoid biosynthesis pathway (Supplementary Figure S7B). Genes co-localized with the DE lncRNAs from T3 vs. D3 were involved in more than 30 metabolic pathways, and three of them (ribosome, photosynthesis and flavonoid biosynthesis) were significantly enriched (Supplementary Figure S7C). The differential expression of lncRNAs between endodormancy and ecodormancy suggested that the lncRNAs are likely involved in the regulation of endodormancy to ecodormancy transition, however, the specific functions of these lncRNAs will need further investigation.

### LncRNA Interactions With microRNA Expression

**FIGURE 7:**
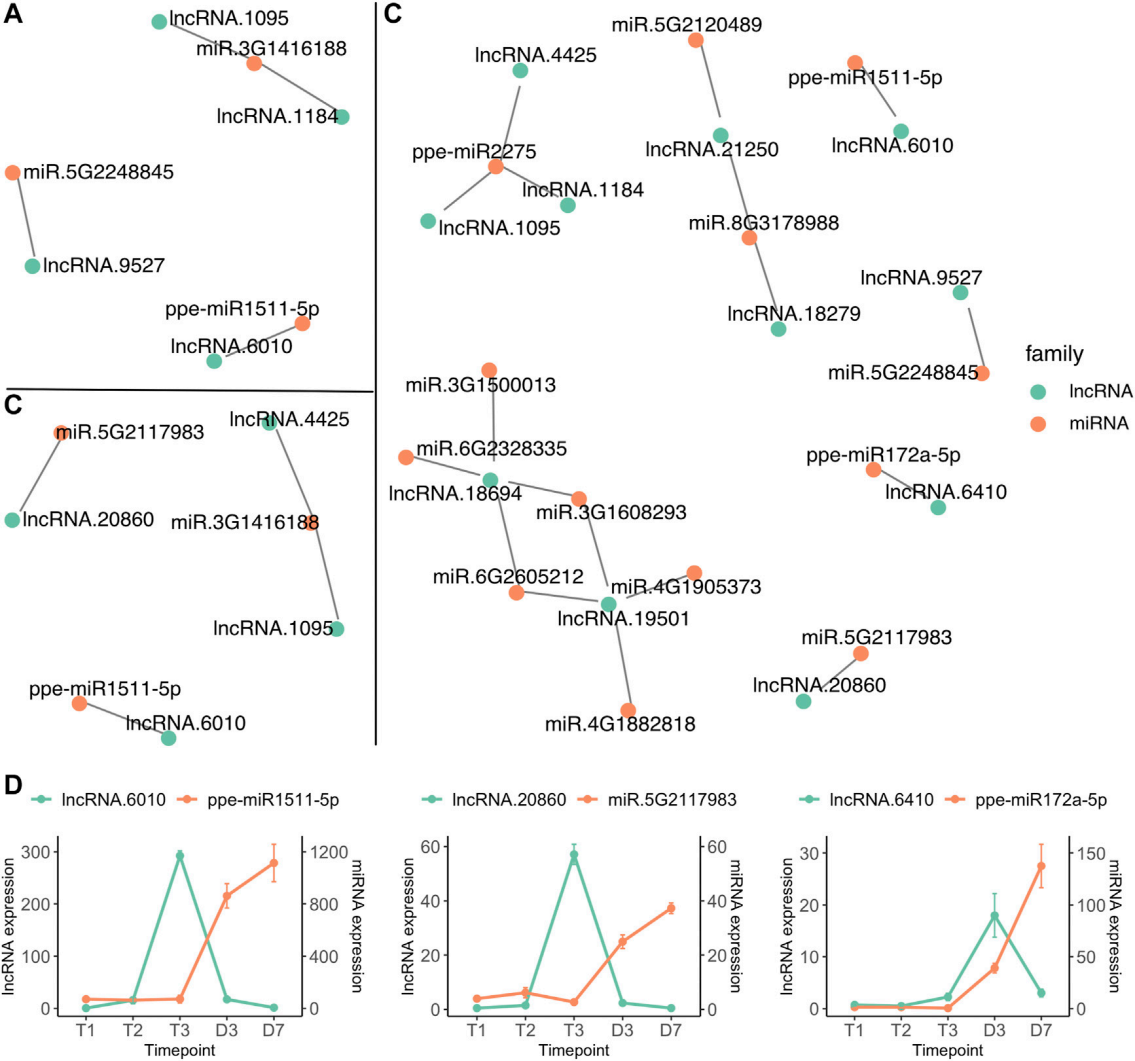
lncRNA (green) and miRNA (red) interaction networks of differentially expressed lncRNAs from T1 vs. T2 **(A)**, T2 vs. T3 **(B)** and T3 vs. D3 **(C) (D)** The expression profiles (mean ± SE) of microRNA and lncRNA pairs that are both differentially expressed between T3 and D3.

Interactions between microRNA and lncRNA have been reported to regulate flower development and reproduction (Zhang et al., 2014b; Wu et al., 2019; Das et al., 2020). LncRNAs can act as microRNA sponges by providing an alternative binding target. This leads to lncRNA expression indirectly influencing the expression of the mRNA normally targeted by the miRNA (Paraskevopoulou and Hatzigeorgiou, 2016). The interactions of microRNAs and lncRNAs were predicted by psRNATarget (Dai et al., 2018), indicating that 88 microRNAs could target 166 sites within 24 lncRNAs. The interactions between microRNAs and lncRNAs from three comparisons, T1 vs. T2, T2 vs. T3 and T3 vs. D3 were investigated. Four lncRNAs differentially expressed between T1 and T2 were identified to have target sites for three microRNAs (Figure 7A), however, none of the microRNAs were differentially expressed between T1 and T2. Two lncRNAs (lncRNA.1095 and lncRNA.4425) upregulated at T3 were predicted to interact with ppe-miR2275 (miR.3G1416188) which was also differentially expressed between endodormancy and ecodormancy (Figure 7B and Figure 4B). 11 DE lncRNAs from T3 vs. D3 were found to interact with 14 microRNAs (Figure 7C). In addition to ppe-miR2275, three pairs of microRNA and lncRNAs were both differentially expressed between T3 and D3 (Figure 7D). We found a consistent expression pattern across the three pairs. The lncRNAs were upregulated earlier than their paired microRNAs, followed by upregulation of the microRNAs while their paired lncRNAs were downregulated (Figure 7D). The opposite expression patterns between the interacted microRNAs and lncRNAs suggest that microRNA may also regulate lncRNA transcript expression. Our findings also suggest a potential function for lncRNA in mediating the activity of microRNAs during dormancy stage transitions.

## Discussion

### microRNAs and lncRNAs Involved in the Regulation of CR and Bud Break

A previous study in chill responsive microRNAs from peach vegetative buds reported that microRNAs and their target genes co-localized with quantitative trait loci (QTL) associated with chill requirement (CR) and bloom data (BD) (Barakat et al., 2012). Using the 10 CR and 10 BD QTL from a peach QTL study (Zhebentyayeva et al., 2014), we found that 12 known microRNAs and 22 novel microRNAs identified in this study are located in the QTL regions. One out of three differentially expressed microRNAs between endodormancy and ecodormancy, miR6285, is co-localized with QTL qCR8-2009. miR6285 has been characterized as a *Prunus* specific microRNA only found in peach, almond and apricot (Gao et al., 2012; Karimi et al., 2016; Niu et al., 2016a). It was also found to be a cold-responsive microRNA in almond and was differentially expressed between cold tolerance and cold sensitive genotypes in both anther and ovary tissues (Karimi et al., 2016). Here we found that ppe-miR6285 and its target gene *NRP* expression profiles not only supported the idea that the miR6285 is responsive to cold stress, but also additionally suggests it as a potential regulator of endodormancy to ecodormancy transition. Of the 100 DE microRNAs from T3 vs. D3 (Figure 2B), eight (3 known and 5 novel microRNAs) were located within the CR QTL and six (1 known and 5 novel microRNAs) were within the BD QTL. Only two microRNAs, miR.5G2240526 on qBD5-2008 and miR8122-5p on qCR7-2009 have degradome supported gene targets also differentially expressed between T3 and D3. However, both these microRNAs were positively correlated with their target genes Prupe.8G088700, encoding a cysteine protease RESPONSIVE TO DEHYDRATION19 (RD19) and a pectin lyase gene Prupe.3G203900, respectively (Supplementary Table S10). RD19 and pectin lyase are both involved in disease resistance (Bernoux et al., 2008; Cao, 2012). By comparing dormant stages and bud break stages, more than half of the microRNAs were positively correlated with their target mRNAs. Positive correlation of expression between microRNAs and their targets has also been observed in other plant species (Lopez-Gomollon et al., 2012; Chen et al., 2015; Cárdenas et al., 2017; Neller et al., 2018; Liang et al., 2019). As the target genes change in expression over developmental stages, additional regulatory factors including transcription factors in tandem with microRNAs are likely involved. Alternatively, these miRNAs may have targets that are not yet identified from the available degradome data.

MicroRNAs are responsive to abiotic stresses in plants, improving plant stress tolerance (Zhang, 2015). The majority of our degradome supported microRNAs targets belong to protein families with enzymatic activities (Supplementary Table S3). Two microRNAs, miR.6G2387859 and ppe-miR393b upregulated at bud break stages (D3 and D7), were identified to target differentially expressed genes encoding transcription factors (Prupe.6G229000 and Prupe.1G173300). Prupe.6G229000 is a MYB73 homolog targeted by microRNA miR.6G2387859 which is upregulated at D3 and D7, however, the expression of Prupe.6G229000 decreased from T2 to D7 (Supplementary Figure S8). MYB73 was co-expressed with CBFs which were induced under cold conditions and it has been shown that MYB73 participated in SA and JA signaling induced by pathogens (Vogel et al., 2005; Jia et al., 2011). The decreased expression levels of MYB73 at ecodormancy and bud break stages suggest that it may be responsive to chilling and then further inhibited by miR.6G2387859 under warm temperatures. Another gene Prupe.1G173300 encodes a basic helix-loop-helix (bHLH) transcription factor targeted by ppe-miR393b (Supplementary Table S3), however, both of the ppe-miR393b and bHLH gene were upregulated at D7 stage (Supplementary Figure S8). In Arabidopsis, overexpression of miR393 inhibited bHLH74 protein and repressed root development in seedlings (Bao et al., 2014). Here we showed that ppe-miR393 was responsive to warm temperatures but not inhibiting the expression of its target gene. Our results suggest that the transcription factors targeted by chill or warm responsive microRNAs are more likely in response to temperatures although microRNAs have potentials mediating transcription factors during dormancy.

A number of lncRNAs significantly induced at ecodormancy were also found to be co-localized with CR QTL regions. LncRNAs can regulate gene expression at different levels. The antisense genic lncRNAs may act as a *cis* regulator to their overlappinging genes (Liu et al., 2015). Recent studies demonstrated that lncRNAs activate or repress gene expressions through regulation of histone modification and protein ubiquitination (Yoon et al., 2013; Kim et al., 2017; Zhao et al., 2018; Zhang et al., 2019) and chromatin loop formation (Ariel et al., 2014). We found that the majority of the 55 DE lncRNAs between T2 and T3 were located in intergenic regions and only half of them are negatively correlated with their neighboring genes (Supplementary Table S8). Nine lncRNAs were on the CR QTL regions (highlighted in red in Supplementary Table S8). One antisense lncRNA.20860 was found upstream of Prupe.8G183600 encodes dihydroflavonol 4-reductase-like1 (DRL1), which is an ortholog of rice anther-specific gene *OsTKPR1* required for pollen wall formation (Xu et al., 2019). Both lncRNA.20860 and Prupe.8G183600 were significantly upregulated at ecodormancy, however, lncRNA.20860 was downregulated at bud break whereas Purpe.8G183600 continued increasing at D3 (Supplementary Figure S9). Their expression profiles suggest that the lncRNA.20860 may be associated with pollen development at ecodormancy, but not at bud break stages. Two other QTL colocalized lncRNAs, lncRNA.482 in qCR1d-2008 and lncRNA.1855 in qCR7-2009, found upstream of cinnamate-4-hydroxylase (Prupe.1G064900) and flavanone 3-hydroxylase (Prupe.7G168300), respectively. Both genes are involved in the flavonoid biosynthesis pathways (Mouradov and Spangenberg, 2014) indicating that lncRNAs may participate in stress-responsive flavonoid pathways at ecodormancy.

### Ppe-miR2275 may be Involved in Pollen Development at Ecodormancy

miR2275 cleavage triggers biogenesis of 24-nt phasiRNA in both monocots and eudicots, and this biogenesis occurs in anthers, specifically initiating at the meiotic stage through the pollen maturation stage (Kakrana et al., 2018; Sun et al., 2018; Xia et al., 2019; Dhaka et al., 2020; Zhang et al., 2020) During dormancy release in peach floral buds, chilling subsequently induces formation of the archesporial cells (anther progenitors) and microsporangium walls where successive meiosis occurs to produce microspores that develop into mature pollen grains before flowering (Reinoso et al., 2002a; Julian et al., 2011). In the floral buds of *Prunus* spp., meiosis in the anther and genes involved in pollen development are also induced at ecodormancy stage onward (Ríos et al., 2013; Julian et al., 2014; Fadón et al., 2019; Yu et al., 2020), indicating the microsporogenesis process is initiated at a late stage of the dormancy/chilling period and completed during the ensuing warm period. ppe-miR2275 is induced at late stage of chilling period/T3/ecodormancy and peaked at warm temperatures/D3 (Figure 4B). It is likely involved in epigenetic regulation of genes that control meiosis and pollen development in peach flower buds. Unlike apple and pear buds which remain largely unchanged during dormancy and rapidly grow at late ecodormancy, peach floral buds still undergo developmental progression during dormancy release or chilling treatment (Yamane et al., 2011; Saito et al., 2015; Yamane et al., 2019). A recent study showed that male meiosis and growth resumption in sweet cherry is not correlated to CR fulfillment but is induced by warm temperatures (Fadón et al., 2020), which is different from peach and apricot in which microsporogenesis and pollen meiosis are determined by CR (Reinoso et al., 2002b; Ríos et al., 2013; Julian et al., 2014). Hence, the detection of ppe-miR2275 upregulated in peach floral buds suggests that it may be involved in the regulation of pollen meiosis initiating at ecodormancy and ensuring development at early bud break.

Our microRNA and lncRNA interaction networks predict that some of the identified microRNAs target DE lncRNAs (Figures 7A–C). For example, ppe-miR2275 is predicted to target three lncRNAs that are significantly induced at ecodormancy (Figure 7C and Supplementary Figure S9). One possible mechanism is that miR2275 targets the lncRNAs to generate phasiRNA, a hypothesis which is also proposed in other plant species (Kakrana et al., 2018). We predicted three putative lncRNAs differentially expressed between ecodormancy and bud break through the computational tool psRNAtarget (Dai et al., 2018), however, only lncRNA.1095 was co-localized with a 24-nt siRNA locus (Supplementary Figure S9). Li et al. (2017) reported that a cassava intergenic lncRNA, lincRNA119, targeted by miR2275, is upregulated with cold stress, and is positively correlated with miR2275 target genes (Li et al., 2017). Further investigations are needed to identify mRNA targeted by ppe-miR2275 and to provide evidence that ppe-miR2275 binds as predicted to the lncRNAs.

### Intronic ncRNA in *DAM4* Intron May Regulate *DAM* Genes

As reported by Zhu et al., this dataset includes reads aligned to the intronic regions of *DAM1* to *DAM6* (Zhu et al., 2020), named as D1ncRNA to D6ncRNA. We also identified these as *DAM* intronic sense non-coding RNAs (intronic lncRNAs D1ncRNA, D2ncRNA, D3ncRNA, D4ncRNA, D5ncRNA and D6ncRNA) transcribed from six *DAM*s (Supplementary Figure S9). Of the six lncRNAs, only D4ncRNA was induced during chill and it peaked at 500 chill hours. The other five lncRNAs had the same expression pattern as their partnered *DAM* genes (Figure 6B). The two known cold-responsive lncRNAs from Arabidopsis, *COLDAIR* and *COOLAIR*, are also transcribed from an intronic region, however, the latter is an antisense lncRNA (Swiezewski et al., 2009; Heo and Sung, 2011; Marquardt et al., 2014). Comparing the expression levels of DAM lncRNAs in our study with the lncRNAs identified in Zhu et al. (2020), Two of the DAM lncRNA (D3ncRNA and D5ncRNA) expression patterns were consistent with the previous study. We found D4ncRNA peaked at T2 (Figure 6B), while in Zhu et al. (2020), D4ncRNA peaked at T3. Although the two studies showed different expression patterns, D4ncRNA was highly expressed during chill hour accumulation in both. As the lncRNA expression levels were detected by regular RNA sequencing in the previous study, the expression levels of D4ncRNA in this study is likely more accurately quantified via strand-specific RNASeq after DESeq2 normalization on the raw counts. *DAM4* from the same experiment was highly expressed at the beginning of dormancy and decreased during dormancy release (Zhu et al., 2020). The expression profiles of D4ncRNA and *DAM4* suggested that the induction of D4ncRNA after chill may repress the expression of *DAM4* during dormancy progression.

### Hc-siRNA Expression Changes During Dormancy and Flowering may Direct Epigenetic Regulation

Hc-siRNA (∼24 nt) is known to induce DNA methylation via the RNA-dependent DNA methylation (RdDM) mechanism (Law and Jacobsen, 2010; Matzke and Mosher, 2014). We identified over 30,000 siRNA loci located in repetitive element regions of the peach genome and classified them as hc-siRNAs. Recent studies suggest that hc-siRNA abundance is positively associated with DNA cytosine methylation of CHH context (Corem et al., 2018; Gyula et al., 2018; Tan et al., 2018). Gyula et al. (2018) found thermoresponsive hc-siRNAs may induce the auxin biosynthesis gene *YUC2* expression via decreasing DNA CHH methylation (Gyula et al., 2018). The majority of hc-siRNA in our study were differentially expressed from T3 to D3 (Figures 5B,C), indicating that hc-siRNA expression may be responsive to warm temperatures. A recent study also showed increased production of 24-nt siRNA targeting *DAM*s from T3 to D3, and such increase concurred with hyper-methylation in CHH context and reinforcement of down-regulation of *DAM*s (Zhu et al., 2020). In sweet cherry, the expression of siRNA and DNA methylation levels in the promoter region of PavMADS1, a gene orthologous to peach *DAM3*, were increased when the chill requirement was fulfilled (Rothkegel et al., 2017). The hc-siRNA co-expression patterns showed about 900 siRNAs were induced at T3 when the chill requirement was fulfilled, which may be involved in regulating the dormancy release (Figures 5B,C). While there is mounting evidence that hc-siRNA induction at late chilling periods/ecodormancy and at flowering provides important epigenetic regulation, further research is needed to identify both the signals that produce these expression changes and how the changes direct floral development and flowering time.

### Non-coding RNAs Associated With Plant Hormones and Secondary Metabolites Contribute to Ecodormancy or Bud Break

Arabidopsis miR167 is required for normal anther and ovule development through negative regulation of auxin response factors ARF6 and ARF8 (Wu et al., 2006; Zheng et al., 2019). Overexpression of ARF6 or ARF8 in Arabidopsis increased auxin response which impaired anther and ovule development, similar to the phenotypes observed in *miR167* mutants (Wu et al., 2006; Yao et al., 2019). The formation of the ovule in peach floral buds occurs at the end of the chill period, near T3, supporting the high levels of miR167 detected in peach flower buds at pre-flowering stages compared to flowering stages. The reduction of miR167a and miR167b from the T2 to T3 stage may play a role in balancing auxin signals to ensure ovule and other floral tissue properly developed (Supplementary Table S11). Jasmonic acid is also involved in meristem and flower development. Jasmonic acid (JA) biosynthesis is regulated by *TEOSINTE BRANCHED/CYCLOIDEA/PCF (TCP)* transcription factors which are targeted by miR319 (Schommer et al., 2008). JA levels and the JA biosynthesis gene transcripts significantly increased during bud break in sweet cherry (Ionescu et al., 2017), poplar (Howe et al., 2015), beech (Juvany et al., 2015), and tea plant (Hao et al., 2017). We identified microRNAs including ppe-miR319 that are differentially expressed between dormancy and bud break stages target biotic and abiotic stress response pathways including response to jasmonic acid stimulus (Supplementary Table S11 and Supplementary Figure S3A). JA is also reported to induce MYC2, which activates the cold stress pathways to enhance cold tolerance (Zhao et al., 2013). The exogenous application of JA can increase Arabidopsis freezing tolerance under cold conditions (Hu et al., 2013). Thus, the JA pathway may contribute to both cold resistance during dormancy and to bud break. The genes in the flanking region of DE lncRNAs involved in flavonoid biosynthesis pathways were also differentially expressed in ecodormancy and bud break stages (Supplementary Table S8 and Supplementary Table S9). Flavonoids are secondary metabolites synthesized from the phenylpropanoid pathway and involved in plant stress responses and development. Our previous work on apricot phenylpropanoids levels during dormancy and flowering showed that phenylpropanoids and related genes may contribute to bud break (Conrad et al., 2019). Flavonoid biosynthesis related genes were differentially expressed between latent buds and lateral buds in grapevine during paradormancy release (Min et al., 2017). Our results indicated that jasmonic acids and flavonoid pathways critical to dormancy release may also be regulated via non-coding RNAs. Thus, regulation of endodormancy release and paradormancy release may share the same metabolic pathways.

## Conclusion

This study provides a genome scale analysis of non-coding regulatory RNAs including small RNAs and long non-coding RNAs during peach bud dormancy release. The expression changes of small RNAs and lncRNAs from endodormancy to bud break suggest that non-coding RNAs play a role in dormancy progression. The majority of non-coding RNAs were differentially expressed between endodormancy and bud break stage, which is more likely in response to the change of temperatures. The lncRNAs and miR2275 associated with pollen meiosis significantly upregulated at ecodormancy agree with genetic and morphological evidence that pollen differentiation is initiated at ecodormancy in peach floral buds. By integrating transcriptome and degradome data with microRNAs and lncRNAs expression profiles, we identified biological pathways such as ABA metabolism, JA biosynthesis, flavonoid biosynthesis, disease resistance and pollen development may be regulated by the microRNAs and lncRNAs during dormancy release. Our findings suggest that noncoding RNAs are an important layer of transcriptional regulation during winter dormancy progression and floral development in peach.

## Data Availability

The datasets presented in this study can be found in online repositories. The names of the repository/repositories and accession number(s) can be found in the article/Supplementary Material.

## Glossary

ABA: abscisic acid
bHLH: basic helix-loop-helix
ARF: auxin response factor
CBF: C-repeat binding factor
CR: chilling requirement
CH: chill hours
DAM: dormancy-associated MADS genes
DE: differential expression
DRL1: dihydroflavonol 4-reductase like 1
FDR: false discover rate
FLC: *FLOWER LOCUS C*
GO: gene ontology
hc-siRNA: heterchromic-small interfering RNA
JA: jasmonate
KEGG: kyoto encyclopedia of genes and genomes
lncRNA: long non-coding RNA
NAT-siRNA: natural antisense transcript small interfering RNA
NCED: 9-cis-epoxycarotenoid dioxygenase
NRP: asparagine-rich protein (NRP)
nt: nucleotides
PARE: parallel analysis of RNA ends
PCA: principal component analysis
PPI: protein-protein interaction
PRC2: polycomb repressive complex 2
QTL: quantitative trait loci
RCA: rubusco activase
ROS: reactive oxygen species
rRNA: ribosomal RNA
siRNA: small interfering RNA
SVK: SVALKA
SVP: *SHORT VEGETATIVE PHASE*
SVL: SVP-like
TCP: *TEOSINTE BRANCHED/CYCLOIDEA/PCF*
tRNA: transfer RNA
WGCNA: weighted gene co-expression network analysis
