# Supplementary material for: Genome-Wide Changes of Regulatory Non-Coding RNAs Reveal Pollen Development Initiated at Ecodormancy in Peach
Source: Front Mol Biosci. 2021 Apr 9;8:612881. doi: 10.3389/fmolb.2021.612881 (PMC8098804; doi:10.3389/fmolb.2021.612881)
Supplement: Supplementary file 1 [file table1.docx]

Supplementary Material

# Supplementary Figures and Tables

Supplementary figures for this study are included in this document. Supplementary tables are included in a separate excel file.

## Supplementary Tables

**Supplementary Table 1**. Total number of reads and mapping rates of each small RNASeq library and strand specific RNASeq library.

**Supplemental Table 2.** The number of differential expression (DE) microRNAs and genes in each comparison.

**Supplementary Table 3.** microRNAs and the degradome supported targets identified in this study.

**Supplementary Table 4.** T1 vs T3 DE genes target by microRNAs.

**Supplementary Table 5.** The number of DE lncRNA in each module.

**Supplementary Table 6**. lncRNA differentially expressed between endodormancy (T1) and ecodormancy (T3). lncRNAs highlighted in red are localized in peach CR QTL.

**Supplementary Table 7.** lncRNA differentially expressed between T1vsT2.

**Supplementary Table 8.** lncRNAs and their neighboring genes differentially expressed between T2 and T3.

**Supplementary Table 9.** lncRNA differentially expressed between ecodormancy (T3) vs bud break (D3).

**Supplementary Table 10.** micrRNA and their target genes differentially expressed between T3 and D3.

**Supplementary Table 11.** Differential expression microRNAs in each comparison with fold change and adjusted p-value.

## Supplementary Figures

**Supplementary Figure 1.** The size distribution of small RNASeq reads.

**Supplementary Figure 2.** Expression frequency of the top 15 most abundant microRNA in each time point. The dash line represented 0.9 of the cumulative expression.

**Supplementary Figure 3.** Co-expression modules detected across the five time points. (A) Module factors relationships between modules and time points. A higher correlation score indicates a higher expression level at the time point. (B) The number of microRNAs in each module.

**Supplementary Figure 4.** microRNA regulated pathways during dormancy release. (A) Gene ontology network of microRNA targeted genes that displayed similar expression profiles. (B) Left: gene interaction network of upregulated microRNAs and their target genes upregulated at bud break. Right: the expression profiles of microRNA (black) and its targeted hub gene (blue) identified by the network on the left. (C) Left: gene interaction network of downregulated microRNA target genes downregulated at bud break. Right: the expression profiles of microRNA (black) and its targeted hub gene (blue) identified by the network on the left.

**Supplementary Figure 5.** Zhu et al. 2020 provided updated structural annotation for DAM1-DAM6 (genome browser track A). Zhu et al also identified intronic ncRNAs in three of those genes, named D3ncRNA, D4ncRNA, D5ncRNA. Based on our manual annotation, we found intronic ncRNAs in the remaining three DAM genes and followed the same naming pattern: D1ncRNA, D2ncRNA, and D6ncRNA (genome browser track B).

**Supplementary Figure 6.** The expression profiles of eigengenes for each lncRNA co-expression module (mean+SE). D1ncRNA, D2ncRNA, and D5ncRNA were clustered into ME5. D4ncRNA and D6ncRNA were clustered into ME4. D3ncRNA was clustered into ME1.

**Supplementary Figure 7**. KEGG pathway analysis of the genes co-localized with DE lncRNAs from T1 versus T2 (A), T2 versus T3 (B) and T3 versus D3 (C). Enrich factor was obtained as the ratio of the number of DE lncRNAs in the pathway and the number of all genes in pathway. Red stars next to the pathways indicated the pathway was significantly enriched with p-values < 0.05

**Supplementary Figure 8.** Expression profiles of two microRNAs and their targeted transcription factors (mean+SE).


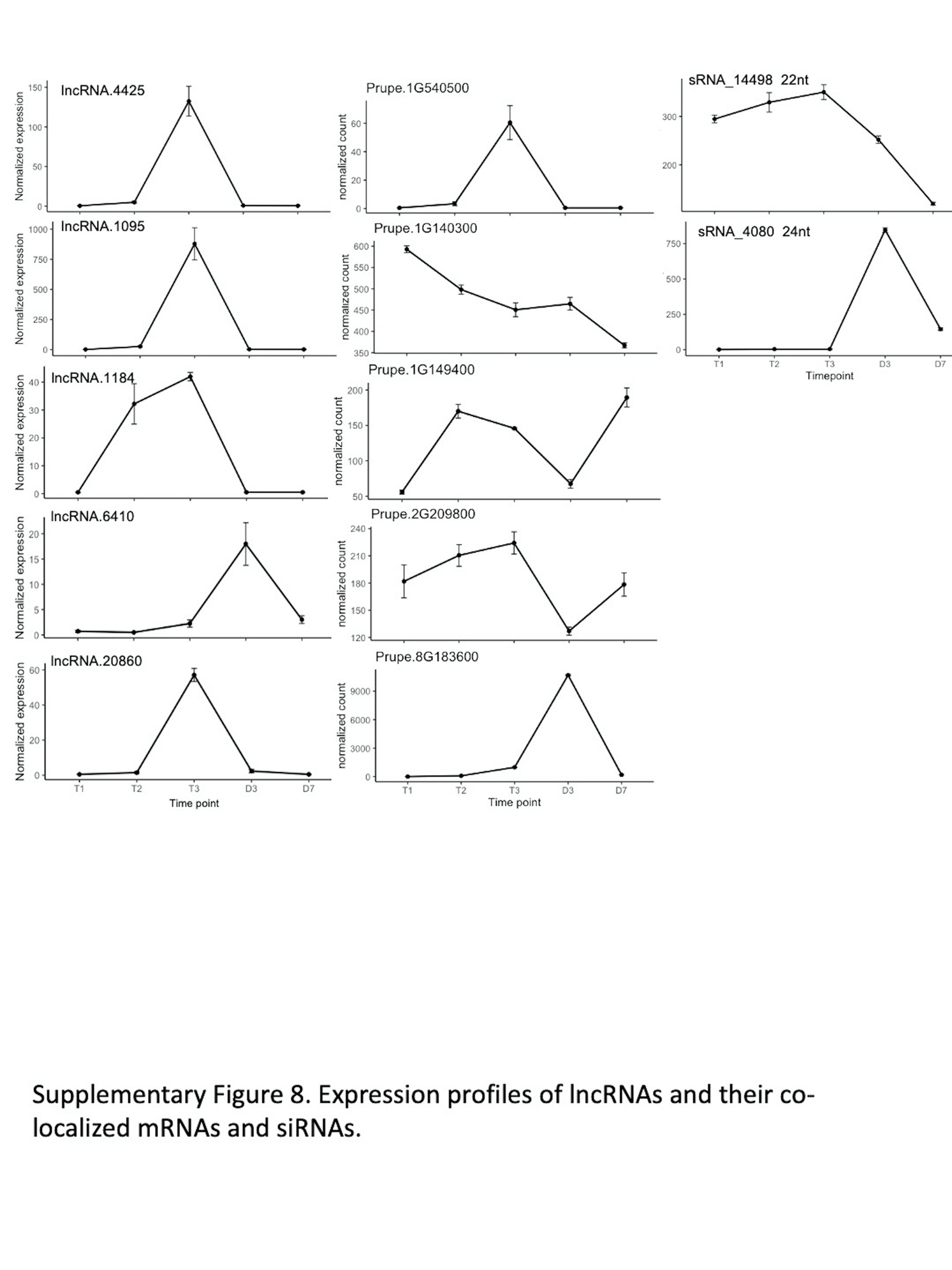


**Supplementary Figure 9.** Expression profiles of lncRNAs and their co-localized mRNAs and siRNAs (mean+SE).
